# Jejunostomy with Enteroenterostomy for Enteral Nutrition in Critically Ill Trauma Patients. A Novel Technique

**DOI:** 10.7759/cureus.3431

**Published:** 2018-10-09

**Authors:** Shahram Paydar, Nader Moein-Vaziri, Maryam Dehghankhalili, Hossein Abdolrahimzaeh, Shahram Bolandparvaz, Hamid Reza Abbasi

**Affiliations:** 1 General Surgery, Shiraz University of Medical Sciences, Shiraz, IRN

**Keywords:** jejunostomy, enteroenterostomy, trauma, enteric nutrition, critically ill patient

## Abstract

Purpose

The aim of the current study was to report the surgical outcome and complications of jejunostomy with enteroenterostomy for enteral nutrition (EN) in critically ill trauma patients with prolonged nasogastric (NG) nutrition.

Methods

This cross-sectional study was carried out in a level I trauma center in Shiraz, southern Iran during a one-year period from 2016 to 2017. We included a total number of 30 patients with severe trauma admitted to the intensive care unit (ICU) with more than three months NG nutrition and bowel atrophy. We performed a novel jejunostomy with an enteroenterostomy procedure for providing a route for enteral nutrition in all 30 patients. The rate of complications, such as dislodgement, clogging, obstruction, leakage, mucosal bleeding, and infection, were recorded and reported. We also recorded the hospital and ICU length of stay (LOS).

Results

We included a total number of 30 patients with a mean age of 35.64 ± 8.91 years, and there were 23 (76.6%) men and seven (23.4%) women among the patients. Overall, 14 (46.6%) patients experienced complications related to the jejunostomy with enteroenterostomy. The most common complication was nausea and vomiting (33.3%) and distention (33.3%), followed by surgical site infection (30.0%). The mean ICU LOS and hospital LOS was found to be 16.8 ± 3.7 and 24.3 ± 4.1 days, respectively. The overall mortality rate was 17 (56.6%), which was secondary to the primary injury and was not related to the procedure.

Conclusion

Jejunostomy with enteroenterostomy is a safe and feasible method for providing a route for EN in critically ill trauma patients with prolonged NG nutrition and bowel atrophy.

## Introduction

Current guidelines all recommend the early enteral nutrition (EN) in critically ill surgical and trauma patients to reduce the infectious complications and the mortality rate through different mechanisms [1-5]. In addition, several lines of evidence suggest that early EN is superior to early parenteral nutrition (PN) in reducing the infection rate, complications, and the mortality, especially in patients with trauma and abdominal injuries [6-8]. Several lines of evidence suggest that prolonged nasogastric nutrition in critically ill patients is associated with bowel atrophy and subsequent complications [9-10]. Currently, early EN is given to all the critically ill patients with traumatic injuries, and this has been associated with improved outcomes and decreased complication rates in different centers worldwide. However, in source-limited hospitals, early jejunostomy is not applicable, especially in patients with severe traumatic brain injury (TBI), mostly because of ethical issues. In these patients, most of the guardians do not consent for early jejunostomy with a hope of early increased level of consciousness and oral feeding [11]. Thus, we usually encounter an atrophic bowel when performing jejunostomy which is, in turn, associated with increased feeding intolerance and a higher incidence of an enterocutaneous fistula after the tube removal [11-12].

The tube jejunostomy was introduced in 1879 by providing an enteral access into the proximal jejunum [13]. Since that time, several advances in the surgical approach to EN feeding tubes have occurred, including the introduction of percutaneous and laparoscopic techniques, as well as gastrostomy tubes with an extension into the jejunum [14]. Currently, several methods, such as longitudinal Witzel [12] and transverse Witzel [11] techniques, are available for surgical tube jejunostomy. The jejunostomy procedure in patients with prolonged NG nutrition and atrophic bowel is associated with several complications secondary to the Lembert sutures used to fix the catheter to the atrophic bowel. We proposed that placing an enteroenterostomy before the jejunostomy will help the passage of gastric, bile, and pancreatic secretions easily, resulting in a decreased complication rate. In the current study, we provide a novel surgical technique which increases the possibility of jejunostomy tunneling and improves the flow of biliary and pancreas secretions in critically ill trauma patients with prolonged NG nutrition and bowel atrophy.

## Materials and methods

Study population

This prospective cross-sectional study was conducted during a one-year period from September 2016 to August 2017, in Rajaei Trauma Hospital, a Level I trauma center in Shiraz, southern Iran, affiliated with the Shiraz University of Medical Sciences. The study protocol was approved by the Institutional Review Board (IRB) and the Medical Ethics Committee of Shiraz University of Medical Sciences (approval #96-01-01-10771). All the patients or their legal guardians provided their informed written consents before inclusion in the study. We included adult (≥ 16 years) critically ill patients, admitted to the intensive care units (ICUs) of our center with a severe traumatic injury who received at least three months of NG nutrition and required jejunostomy. All the patients had severe bowel atrophy. We excluded those with severe bowel injuries distal to the ligament of Treitz, bowel obstruction, short bowel syndrome, high-output fistula, very severe Crohn's disease or ulcerative colitis, patients with ascites, terminally ill patients, and those who did not provide their consents to be included. 

Study protocol

All the eligible patients were evaluated preoperatively regarding the hemostasis and blood factors. The baseline characteristics, including the demographics, mechanism of injury, comorbidities, injuries to the other organs, injury severity score (ISS) and clinical parameters, were recorded. All the patients underwent surgical placement of a jejunostomy with enteroenterostomy in our center with general anesthesia and by the same team of the surgeons. The patients received EN after 72 hours of operation. They were all followed and evaluated by the general surgery residents and also in the outpatient clinics. We recorded the infection at the insertion site, intra/retroperitoneal feed leakage, nausea, paralytic ileus, feed-associated diarrhea, abdominal cramps, and delayed gastric emptying. We also recorded the survival, ventilator days, hospital length of stay (LOS), ICU LOS, and ventilator-associated pneumonia.

Surgical procedure

**Figure 1:**
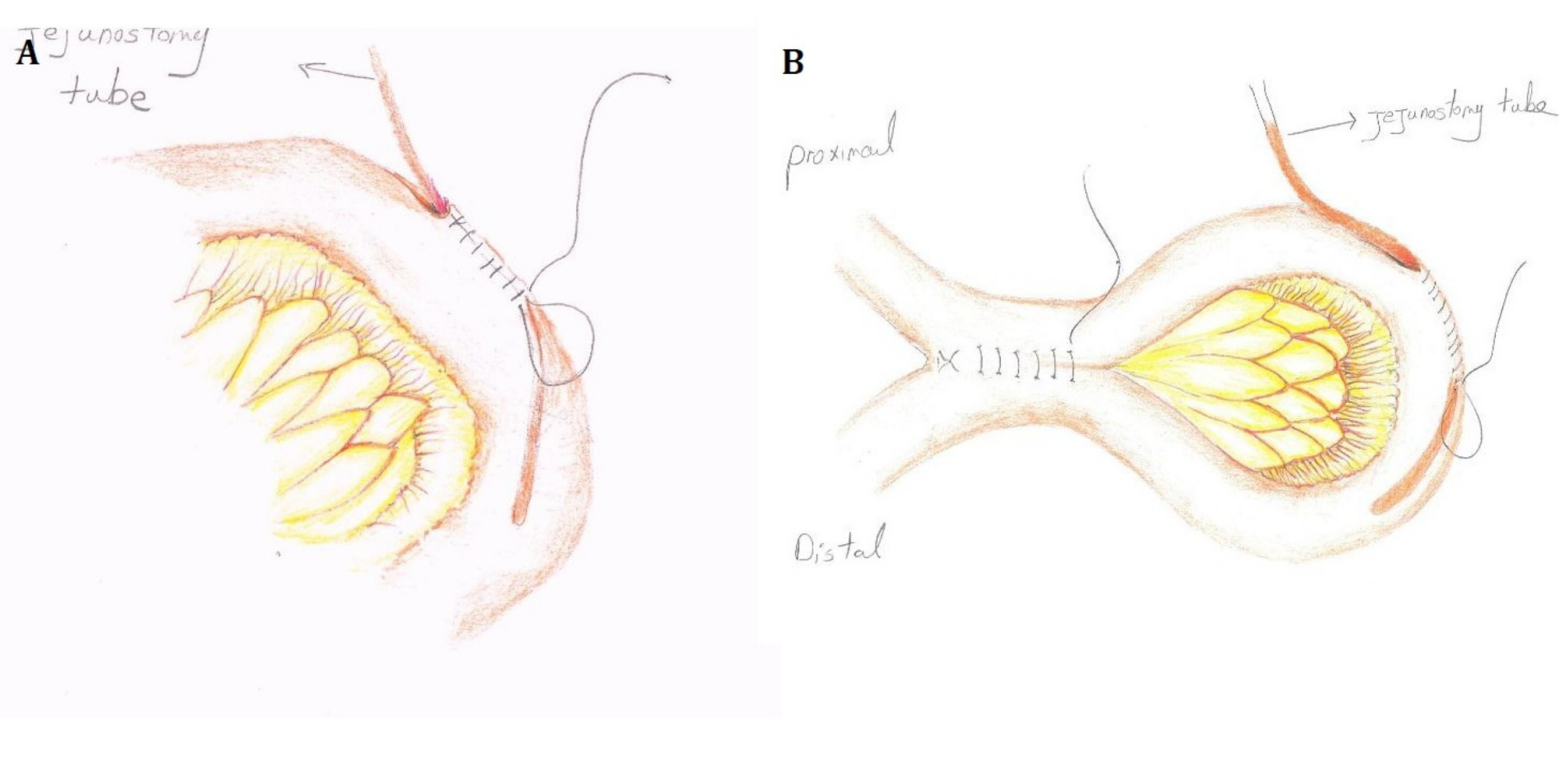
Schematic View of the Jejunostomy with Proximal Enteroenterostomy A) First, the bowel loop, 30-cm distal to the ligament of Treitz, is pierced and the tube is inserted; B) the proximal loop is then grabbed and the enteroenterostomy is performed

The peritoneum was entered via a midline incision. The bowel loop was grabbed using Babcock forceps. A small stab wound was made on the antimesenteric border of the jejunum, and a rubber tube was inserted distally for approximately 30 cm. A purse-string suture was placed around the tube at the jejunostomy site (Figure 1A). Enterostomy sites were 1 cm longitudinal and located 15 cm proximal and distal to the jejunostomy site. The enteroenterostomy was done in two layers using Prolene™ (Ethicon, Inc., Cornelia, GA) and polydioxanone (PDS) 3-0 suturing material (Figure 1B). The jejunostomy tube was placed in the seromuscular tunnel which was about 10 cm longitudinal; the tunnel was then closed with interrupted sutures, and the tube was brought out through the anterior abdominal wall via a separate incision in the left upper quadrant of the abdomen. The jejunum was sutured to the abdominal wall at the end of the procedure [12].

Statistical analysis

All the data were analyzed using the Statistical Package for Social Sciences (SPSS) (IBM SPSS Statistics, Armonk, NY), version 20.0. All the data are presented as the mean ± standard deviation (SD) and proportions as appropriate.

## Results

Overall, we included a total number of 30 critically ill trauma patients with prolonged NG nutrition and bowel atrophy who underwent jejunostomy with enteroenterostomy in our center during the study period. The mean age of the patients was 37.6 ± 11.6 (range: 18 to 56) years. There were 23 (76.6%) men and seven (23.4%) women among the patients. Motor vehicle accidents were the most common mechanism of injury recorded in 26 (86.7%) patients. The mean ISS was 23.37 ± 4.6 (range: 18 to 37), and traumatic brain injury (TBI) was the most common co-injury recorded in 20 (66.6%) patients. The baseline characteristics are summarized in Table 1.

**Table 1:** The Baseline Characteristics of 30 Critically Ill Trauma Patients Undergoing Jejunostomy with Enteroenterostomy in Our Center During the Study Period

Value	Variable
Age (years)	37.6 ± 11.6
Gender	
Men (%)	23 (76.6%)
Women (%)	7 (23.4%)
Mechanism of injury	
Motor-vehicle accident (%)	26 (86.7%)
Fall (%)	3 (10.0%)
Assault (%)	1 (3.3%)
Injury Severity Score	23.37 ± 4.6
Organ injuries	
Neurosurgery (%)	20 (66.6%)
Orthopedics (%)	13 (43.3%)
Lung (%)	9 (30.0%)
Abdomen (%)	1 (3.3%)
Previous surgeries	
Laparotomy (%)	2 (6.6%)
Others (%)	8 (26.6%)

Overall, 14 (46.6%) patients experienced complications related to the jejunostomy with enteroenterostomy. None of the patients developed enterocutaneous fistulas after tube removal. The most common complication was nausea and vomiting (33.3%) and distention (33.3%), followed by surgical site infection (30.0%) and tube re-insertion (16.6%). The mean ICU LOS and hospital LOS was found to be 16.8 ± 3.7 (range: 9 to 28) and 24.3 ± 4.1 (range: 14 to 36) days, respectively. The ventilator-dependent duration was 4.83 ± 2.3 (range: 2 to 8) days, while only four patients (13.3%) were reported to suffer from ventilator-associated pneumonia. The overall mortality rate was 17 (56.6%), which was secondary to the primary injury and was not related to the procedure. The outcome of the 30 patients included in the current study is summarized in Table 2.

**Table 2:** The Outcome of 30 Critically Ill Trauma Patients Undergoing Jejunostomy with Enteroenterostomy in Our Center During the Study Period ICU: intensive care unit

Value	Variable
ICU length of stay (days)	16.8 ± 3.7
Hospital length of stay (days)	24.3 ± 4.1
Ventilator duration (days)	4.83 ± 2.3
Ventilator-associated pneumonia (%)	4 (13.3%)
Mortality (%)	17 (56.6%)
Complications	
Nausea and vomiting (%)	10 (33.3%)
Distention (%)	10 (33.3%)
Surgical site infections (%)	9 (30.0%)
Tube re-insertion (%)	5 (16.6%)
Feeding intolerance (%)	4 (13.3%)
Electrolyte imbalance (%)	4 (13.3%)
Diarrhea (%)	3 (10.0%)
Metabolic complications (%)	3 (10.0%)
Bowel obstruction (%)	2 (3.33%)

## Discussion

In the current study, we have introduced a novel and innovative technique for reducing complications related to the jejunostomy for providing a route for EN in those critically ill trauma patients receiving prolonged NG nutrition and subsequent bowel atrophy. Actually, Lambert sutures are used to fix the jejunostomy tube to the bowel wall. In patients with prolonged NG nutrition and atrophic bowel, the Lambert sutures cause the lumen to be narrowed and thus the secretions of the gastric mucosa, bile, and pancreas congregate before the Lambert sutures. We proposed that adding an enteroenterostomy before the jejunostomy site will result in free passages of these secretions and thus increasing the bowel tolerance and reducing the complications. We have demonstrated that this method is a safe and feasible technique and associated with minimal complications and side effects. None of the patients developed enterocutaneous fistula after removing of the tube. Thus, we recommend this technique, especially in critically ill trauma patients. However, further randomized clinical trials are required to shed light on the efficacy and adequacy of this technique.  

The benefit of early EN therapy for critically ill trauma patients is well-established [1-2, 6, 15]. However, early jejunal feeding for patients who require major intestinal surgery or have severe intestinal injuries is not without risk. Patients with severe intestinal injuries are more likely to experience feeding jejunostomy-associated complications, such as intestinal leaks, perforations, volvuli, secondary infections, and bowel necrosis [16]. In addition, in developing countries (like Iran) with source-limited hospitals, early jejunostomy is usually not allowed by the guardians with the hope of an early increased level of consciousness and starting the oral feeding. This results in severe bowel atrophy which, in turn, causes a more difficult procedure with higher complication rates, especially the feeding intolerance and the enterocutaneous fistula [11-12]. The complications related to the jejunostomy include major complications (such as small bowel perforations, small bowel volvuli with infarction, intraperitoneal leaks, and small bowel necrosis) and minor ones (such as nausea and vomiting, distention, surgical site infection, feeding intolerance, and diarrhea) [17-18]. Diligent and meticulous monitoring of the critically ill patient with severe intestinal trauma and jejunostomy feeding is mandatory.

In the current study, we did not experience any major complication. We recorded only two bowel obstructions, which were managed conservatively, and no other surgical intervention was required. About half of the patients experienced minor complications associated with the jejunostomy, including nausea and vomiting, diarrhea, distention, and surgical site infection. There was no mortality associated with the procedure, while the Witzel method is associated with 1.4% to 5% mortality [16, 19-20]. Further studies are required to demonstrate the long-term outcomes of the procedure and its nutritional effects.

We note some limitations to our study. First, we included a limited number of patients in this study. This is because this was a pilot study and we wanted to evaluate the short-term and surgical complications of this technique. Larger studies are now underway in our center for providing the appropriate evidence. Second, we did not include a group of patients undergoing jejunostomy with the Witzel method alone. This limitation does not let us discuss the superiority or inferiority of this novel technique compared to the standard method. Lastly, we did not measure the nutritional indices before and after the procedure and thus cannot comment on the nutritional adequacy of the procedure. However, this is a case series reporting the outcome of a novel technique of jejunostomy with proximal enteroenterostomy for EN in critically ill trauma patients.

## Conclusions

Jejunostomy with enteroenterostomy is a safe and feasible method for providing a route for EN in critically ill trauma patients with prolonged NG nutrition and severe bowel atrophy. The results of the current cross-sectional study demonstrated that this novel technique is associated with acceptable and favorable outcomes and minimal complication rates in critically ill trauma patients. Further comparative studies with a standard method are required for providing the evidence accordingly.
